# Renin Kinetics and Hemodynamic Response During Treatment of Vasoplegic Shock With Angiotensin II (TENSINTEST): Protocol for a Multicenter Observational Study

**DOI:** 10.2196/102437

**Published:** 2026-11-09

**Authors:** Andreja Möller Petrun, Katarina Barbara Bajec, Lakhmir Chawla, Jan Zmazek, Andrej Markota

**Affiliations:** 1 Department of Anaesthesiology, Intensive Therapy and Pain Management, University Clinical Centre Maribor, Ljubljanska 5, Maribor, Maribor City Municipality, Slovenia, 386 23211568; 2 Faculty of Medicine, University of Maribor, Maribor, Maribor City Municipality, Slovenia; 3 Veterans Affairs Medical CenterSan Diego, CA, United States; 4 Faculty of Natural Sciences and Mathematics, University of Maribor, Maribor, Maribor City Municipality, Slovenia; 5 Medical Intensive Care Unit, University Clinical Centre Maribor, Maribor, Maribor City Municipality, Slovenia

**Keywords:** shock, distributive, angiotensin II, renin, vasoplegia

## Abstract

**Background:**

Shock is a life-threatening condition that can lead to multiorgan failure and death. Vasopressors are needed for the treatment of vasoplegic (distributive) shock. Angiotensin II (AT II) is a novel vasopressor. Approximately two-thirds of patients respond to AT II, and one-third do not. AT II is part of the renin-angiotensin-aldosterone system (RAAS). Renin is the limiting enzyme of the RAAS, and among other mechanisms, its concentration is regulated by AT II concentration through a negative feedback loop. It might be an indicator of response to AT II therapy.

**Objective:**

We designed a study with a primary objective of developing and standardizing an AT II stress test, which could help predict the responsiveness to AT II in patients with distributive shock, regardless of its origin.

**Methods:**

This is an investigator-initiated, multicenter observational clinical study of renin kinetics before and 6 hours after starting an infusion of AT II in patients with vasoplegic shock and adequate fluid resuscitation who are severely ill. Prior to AT II, patients will receive an infusion of norepinephrine base of 0.3 mcg per kilogram per minute or higher, and vasopressin of 0.03 IU per minute according to institutional protocol. Blood samples for serum renin determination will be obtained immediately prior to and after 6 hours of AT II infusion. We hypothesize that patients who demonstrate a negative trend in relative renin ratio (defined as renin plasma concentration at baseline or at 6 hours after treatment with AT II divided by upper normal concentration of plasma renin [50 mU/L]) will have a greater number of 28-day organ failure–free days (days without vasopressors, mechanical ventilation, or renal replacement therapy). The primary analysis will assess the association between early renin reduction and organ failure–free days using an adjusted proportional odds ordinal regression model.

**Results:**

The research was funded in December 2024. After obtaining ethics approval in July 2025, data collection started on August 1, 2025. As of July 2026, a total of 70 patients had been enrolled. Data collection is expected to be completed in 2027, after which the results will be analyzed and published.

**Conclusions:**

The goal of our study is to determine whether renin 0-6 h kinetics (difference between renin levels before and 6 hours after starting an AT II infusion) are associated with clinical outcomes in patients who require AT II. Our study could contribute to the development of a more individualized approach to vasopressor therapy and avoidance of inappropriate use of AT II in patients who are not responders, allowing earlier discontinuation of AT II and consideration of alternative therapies.

## Introduction

Shock is a life-threatening condition leading to multiorgan failure and death from inadequate oxygen and nutrient delivery to cells [1]. Septic shock, the most common form of distributive shock, is managed with norepinephrine as the first-line vasopressor, with vasopressin recommended as an adjunct in patients requiring escalating catecholamine support [2,3]. Although this strategy is effective for many patients, it follows a largely uniform approach despite growing evidence that vasodilatory shock is biologically heterogeneous, and there are limited data on other vasopressors, which underscores the need for personalized therapy [1,4,5]. Angiotensin II (AT II) exemplifies this as approximately two-thirds of patients achieve the target mean arterial pressure (MAP) following AT II initiation, while nearly one-third do not [6,7]. There are also very little data on how to predict responsiveness to AT II before the infusion [8-10], emphasizing the need for biomarkers capable of identifying patients most likely to benefit from AT II therapy. AT II is the principal effector peptide of the renin-angiotensin-aldosterone system (RAAS). The RAAS features opposing classic and nonclassic pathways that are balanced in health but dysregulated in vasodilatory shock, showing inappropriate nonclassic activation [11-13]. As shown by Strik-Lips et al [14], AT II infusion can restore physiological RAAS negative feedback but only in hemodynamic responders, as evidenced by a rapid decline in plasma renin concentrations, whereas nonresponders exhibited persistent renin elevation despite higher circulating AT II levels and demonstrated activation of the alternative RAAS pathway. Renin, the rate-limiting enzyme for both RAAS pathways, is released by the kidneys during volume depletion, hypotension, hypoxia, or sympathetic activation, with negative feedback from AT II therefore emerging as a promising functional biomarker of RAAS integrity [15-17]. Studies associate reduced levels of renin after AT II (eg, after cardiac surgery) and elevated baseline renin (three-fold above normal) with better responses in distributive shock, marked by higher MAP and lower vasopressor needs [18-20]. Renin also predicts severity of illness, outperforming lactate for mortality and development of acute kidney injury [21,22].

This study aims to develop and standardize an AT II stress test to predict outcomes in patients with distributive shock regardless of origin. It assesses renin levels before and 6 hours after AT II initiation vs clinical outcomes, with the primary hypothesis that the negative trend in relative renin ratio (defined as renin plasma concentration at baseline or at 6 hours after treatment with AT II divided by the upper normal concentration of plasma renin [50 mU/L]) 6 hours after starting AT II infusion is associated with a higher number of 28-day organ failure–free days (days without vasopressor therapy, invasive mechanical ventilation, or renal replacement therapy) and a higher number of 28-day intensive care unit (ICU)–free days. We further hypothesize that the negative trend in renin kinetics reflects the clinically observed responses to AT II and is associated with reduced vasopressor requirements and lower mortality.

The results aim to enable individualized vasopressor therapy by helping clinicians decide whether treatment with AT II should be continued. A negative trend in the relative renin ratio may help identify patients who are responding to AT II and who are more likely to have improved clinical outcomes (expected gains in 28-day organ failure–free days and shorter ICU stays). Conversely, in patients who fail to exhibit a negative trend in the relative renin ratio with unfavorable hemodynamic response, continued AT II therapy could be reconsidered, and alternative vasopressor or adjunctive therapies could be sought.

## Methods

### Trial Design

Angiotensin II Stress Test (TENSINTEST) is an investigator-initiated, multicenter observational study on renin kinetics before and 6 hours after starting an infusion of AT II in patients with vasoplegic shock.

### Trial Setting

TENSINTEST will be carried out in 8 different ICUs (surgical and medical) in Slovenia. Multimedia Appendix 1 provides details on the included ICUs.

### Eligibility Criteria

All patients aged 18 years or older who are admitted to the ICU because of vasodilatory (distributive) shock regardless of etiology and who fail to achieve an adequate MAP of 65 to 85 mm Hg despite optimal fluid resuscitation and receiving norepinephrine base at a dose of 0.3 mcg per kilogram per minute or higher and vasopressin at 0.03 IU per minute will be enrolled.

Additional inclusion criteria will be the absence of AT II administration before enrollment, an expected survival of more than 24 hours, and no predefined limitations to active treatment.

The exclusion criteria are listed in Textbox 1.

Textbox 1. **Exclusion criteria.**

- Burns affecting more than 20% of the total body surface area
- Acute coronary syndrome
- Bronchospasm unresponsive to conventional treatment
- Liver disease with Model for End-Stage Liver Disease score of ≥30
- Severe active bleeding (anticipated requirement of ≥4 units of packed red blood cells)
- Acute mesenteric ischemia
- Aortic dissection
- Leukopenia of <1000/mm^3^
- Pregnancy
- Raynaud phenomenon and vasospastic disorders
- Active venous or arterial thromboembolic disease
- Systemic sclerosis
- Chronic corticosteroid therapy with a daily equivalent dose of ≥500 mg of hydrocortisone
- Extracorporeal membrane oxygenation
- Cardiac index <2.3 L/min/m^2^ as determined via any method (ultrasound or invasive monitoring)
- Central venous oxygen saturation below 70%

### Intervention

The intervention is designed to test which patients could benefit from AT II infusion. The decision to treat patients with AT II infusion will be made by a treating clinical intensivist as per institutional protocol. The only intervention will be to obtain a sample of blood immediately before and 6 hours after starting AT II infusion to determine the renin concentration. Two additional venous blood samples for measurement of direct (active) renin concentration will be collected into ethylenediaminetetraacetic acid (EDTA) tubes in accordance with the specimen requirements of the LIAISON Direct Renin assay (DiaSorin S.p.A) [23]. Patients will be treated according to established guidelines and protocols in all other aspects of their ICU stay.

### Protocol for the Study

Patients who fulfill the inclusion criteria, meet none of the exclusion criteria, and provide informed consent will be enrolled in the study.

Patients who fail to achieve adequate MAP despite optimal fluid resuscitation, a norepinephrine base of 0.3 mcg per kilogram per minute or higher and vasopressin of 0.03 IU per minute will receive AT II infusion initiated at 20 ng per kilogram per minute as per the institutional protocol for initiation of a third vasopressor when adequate MAP is not achieved (Figure 1).

**Figure 1.**
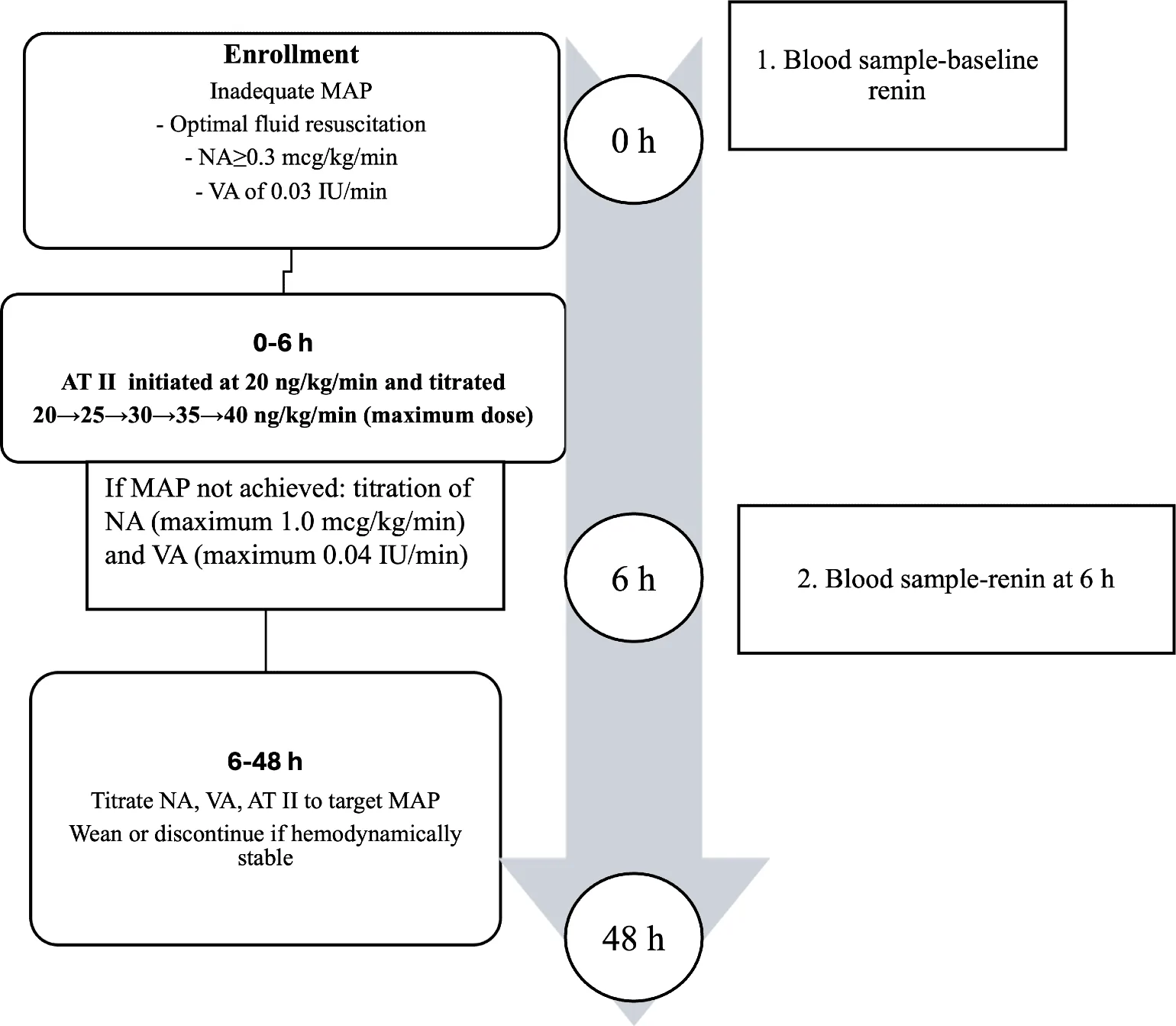
Study design. AT II: angiotensin II; MAP: mean arterial pressure; NA: norepinephrine base; VA: vasopressin.

Adequate fluid resuscitation will include patients who have received an initial crystalloid bolus of approximately 30 mL per kilogram when clinically appropriate, followed by individualized fluid management based on repeated assessment of fluid responsiveness, clinical evaluation, and the treating physician’s judgment.

Following initiation of AT II at 20 ng per kilogram per minute, if the target MAP (65‐85 mm Hg) is not achieved within 15 minutes, the AT II dose will be increased in 5 ng per kilogram per minute increments every 15 minutes up to a maximum dose of 40 ng per kilogram per minute. If the target MAP is still not achieved, norepinephrine and vasopressin may be titrated as needed up to a maximum norepinephrine dose of 1 μg per kilogram per minute and a maximum vasopressin dose of 0.04 IU per minute. If MAP remains insufficient, further adjustments of vasopressors and adjunctive therapies (eg, higher doses of norepinephrine base, methylene blue, etc) will be made at the discretion of the treating physician. The decision to initiate hydrocortisone prior to AT II infusion will also be left to the clinical judgment of the treating physician based on the underlying disease.

In the event of hypertension, norepinephrine base and vasopressin will be reduced and discontinued if necessary. If both are discontinued, then AT II will be reduced in decrements of 5 ng per kilogram per minute to achieve target MAP and discontinued if necessary.

Blood samples for serum renin determination will be obtained immediately prior to and 6 hours after the start of AT II infusion. Renin analyses will be performed at the central laboratory of University Medical Centre (UMC) Maribor. Samples obtained outside UMC Maribor will be centrifuged immediately at the local laboratory, frozen to –85 °C to await transport, and transported at –20 °C to UMC Maribor. All other laboratory analyses will be performed at the local laboratories of participating centers.

Renin concentration in plasma samples will be measured as direct (active) renin using the chemiluminescent immunoassay method on a LIAISON fully automated analyzer platform. EDTA plasma is the validated specimen type for this assay. In the LIAISON Direct Renin assay, renin is sandwiched between a capture antibody adsorbed on magnetizable beads and a second isoluminol-linked antibody that detects the reaction. The relative light units developed by the mixture when exposed to an essential environment are proportional to the concentration of renin and allow its quantification. Renin concentration is expressed in mIU per liter (μIU/mL), with a normal range of 4 to 50 mIU/L and an upper reported limit at a value of 500 mIU/L. For the purpose of the study, the sample will be additionally diluted, and a higher value will be determined if the first result of the analysis is above 500 mIU/L to capture potential renin kinetics above this normally used upper limit. In the results, the values of renin will be given as a relative renin ratio (Multimedia Appendix 1). As renin secretion is influenced by sodium and extracellular volume status, these factors represent potential biological sources of variability in renin concentrations [24]. However, all patients will be enrolled only after adequate fluid resuscitation, and fluid balance will be prospectively recorded during the study period.

In the event of an allergic reaction or other serious adverse event (thrombosis or severe ischemia of the extremities or gastrointestinal tract), AT II will be immediately discontinued, and the patient will receive appropriate treatment in accordance with established clinical guidelines. Mechanical ventilation, renal replacement therapy, antibiotics, and all other routine intensive care treatment will be provided outside of the parameters of the study intervention as per international guidelines and protocols.

Participants will not be excluded from the study after enrollment except in the case of withdrawal of informed consent. In the event of an adverse event, in which treatment with AT II would be discontinued, the reason for treatment discontinuation and the adverse event will be documented in detail, participants will remain in the study, and their available data will be included in the analyses.

### Safety Monitoring

All included patients will be monitored daily for adverse events. As many of the potential adverse events are common complications of vasoplegia or critical illness, additionally, we will monitor prespecified adverse reactions listed in the European Medicines Agency prescribing information for AT II that occur only after recruitment: tachycardia, lactic acidosis, new venous or arterial thrombosis, thrombocytopenia, peripheral digital or limb ischemia, gut ischemia, and new arrhythmia [20].

### Minimization of Bias

Due to the nature of the study, investigators and other ICU staff cannot be blinded. All analyses are preplanned and will be performed by an independent investigator.

### Screening, Study Visits, and Data Collection

Patients with vasoplegia and an infusion of norepinephrine base of 0.3 mcg per kilogram per minute or higher and vasopressin of 0.03 IU per minute will be screened for eligibility by study investigators.

The characteristics of screened patients meeting the inclusion criteria will be recorded as follows: date of screening, age, possible cause of vasoplegia, and the reasons for not being enrolled. All recorded data will be stored in a screening database maintained by the research coordinators and stored on a dedicated and secured computer according to the principles of the General Data Protection Regulation.

For enrolled patients, we will collect demographic data as follows: age, gender, height, weight, comorbidities, therapy before critical illness (including the use of angiotensin-converting enzyme inhibitors and angiotensin receptor blockers), therapy during the ICU stay, and cause of vasoplegia. We will use the results of standard daily laboratory examinations, including complete blood count, electrolytes, C-reactive protein, procalcitonin, interleukin-6, troponin, creatinine, and liver function tests. Additionally, we will collect data on daily fluid balance for the first 48 hours after starting AT II infusion; the need for mechanical ventilation (invasive or noninvasive) and its duration; the need for renal replacement therapy and potential use of cytokine removal membranes; and the need for antimicrobial therapy, results of microbiological cultures, and source of infection.

A schedule of enrollment, interventions, and assessments is shown in Table 1.

**Table 1.** The schedule of enrollment, interventions, and assessments.

	Time point																						
	Enrollment (–T0^a^)	T0	1 h	2 h	3 h	4 h	5 h	6 h	9 h	12 h	15 h	18 h	24 h	27 h	30 h	33 h	36 h	39 h	42 h	45 h	48 h	Each day at the ICU^b^	After discharge from hospital
Enrollment																							
Eligibility screen	✓																						
Informed consent	✓																						
Intervention																							
Renin concentration		✓						✓															
Assessments																							
MAP^c^, norepinephrine base, vasopressin, and AT II^d^		✓	✓	✓	✓	✓	✓	✓	✓	✓	✓	✓	✓	✓	✓	✓	✓	✓	✓	✓	✓	✓	
Fluid balance		✓											✓								✓	✓	
Lactate, PO_2,_ and FiO_2_^e^		✓						✓		✓		✓	✓		✓		✓		✓		✓	✓	
CBC^f^, CRP^g^, PCT^h^, IL-6^i^, liver function test, urea, creatinine, myoglobin, and troponin		✓											✓										
Renal replacement therapy		✓											✓								✓	✓	✓
Mechanical ventilation		✓											✓								✓	✓	
Need for hydrocortisone		✓											✓								✓	✓	
Need for antibiotics		✓											✓								✓	✓	✓
Survival																						✓	✓

a Time point before angiotensin II infusion.

b ICU: intensive care unit.

c MAP: mean arterial pressure.

d AT II: angiotensin II.

e FiO_2_: fraction of inspired oxygen.

f CBC: complete blood count.

g CRP: C-reactive protein.

h PCT: procalcitonin.

i IL-6: interleukin-6.

After completion and validation of data collection, the norepinephrine-equivalent dose (NED) will be calculated from the recorded doses of norepinephrine, vasopressin, and AT II at each predefined time point (0, 1, and 6 hours) using the conversion outlined in Table 2. In the case of the use of an additional vasopressor administered (eg, methylene blue), its use and NED using the conversion scale by Kotani et al [25] will be recorded. The NED will be used in the statistical analyses.

**Table 2.** Conversion of vasopressors to norepinephrine-equivalent dose.

Drug equivalent	Dose	Norepinephrine dose (μg/kg/min)	Conversion scale as suggested by Kotani et al [25]
Norepinephrine	0.1 μg/kg/min	0.1	—^a^
Vasopressin	0.04 U/min	0.1	2.5× vasopressin (U/min)
AT^b^	20 ng/kg/min	0.05	0.0025× AT (ng/kg/min)

a Not applicable.

b AT: angiotensin.

### Statistical Methods

#### Sample Size Calculation

No previous study has directly quantified the association between the 0- to 6-hour change in relative renin ratio and 28-day organ failure–free days. Consequently, the parameters required for a formal sample size calculation for the proposed adjusted proportional odds ordinal logistic regression model, including the expected distribution of organ failure–free days and the anticipated common odds ratio, are not available. The sample size estimate was therefore based on the best available indirect evidence. Previous studies of renin kinetics and related clinical outcomes suggest associations in the low to moderate range. In a substudy of the ATHOS-3 (Angiotensin II for the Treatment of High-Output Shock 3) study [13,19], AT II produced a 54% median renin reduction at 3 hours, which was associated with lower 28-day mortality, corresponding to an approximate correlation of *r*≈0.23 between renin response and survival. In a subsequent cohort [26], each 10-fold higher renin concentration at 24 hours was associated with 7 fewer days alive and renal replacement therapy free, corresponding to *r*≈0.35. In addition, Lee et al [27] reported correlations of *r*=0.40 between plasma renin and Sequential Organ Failure Assessment score and *r*=0.11 between plasma renin and ICU length of stay, suggesting that associations with organ dysfunction are typically in the low to moderate range. Considering these data, a planning correlation of *r*=0.30 was estimated. Using the Fisher *z* method (2-sided α=.05), 85 patients provide 80% power to detect *r*=0.30. Because the primary exposure requires both baseline and 6-hour renin measurements, a conservative allowance of approximately 30% was made for participants in whom the 0- to 6-hour change in relative renin ratio may not be calculable. Reasons may include death before the 6-hour measurement, withdrawal of consent, failure to obtain a study sample, or logistical or laboratory failure. A total of 121 participants are therefore planned to be included, which is expected to provide approximately 85 participants with evaluable paired renin measurements.

#### Statistical Analysis

Descriptive statistics will be used to summarize baseline characteristics, with continuous variables reported as means with SDs or medians with IQRs depending on normality assessed using the Shapiro-Wilk test. Categorical variables will be presented as counts and percentages and compared using the chi-square or Fisher exact test as appropriate.

The primary analysis will assess the association between early renin reduction (within 6 hours of AT II administration) and organ failure–free days. The association will be tested using a multivariable proportional odds ordinal logistic regression model, with higher outcome categories indicating more organ failure–free days. The 0- to 6-hour change in relative renin ratio will be analyzed continuously and scaled to 1 SD. The model will adjust a priori for baseline relative renin ratio, APACHE II (Acute Physiology and Chronic Health Evaluation II) score, and baseline lactate. The adjusted common odds ratio and 95% CI will be the primary effect estimates. The proportional odds assumption will be assessed; if materially violated, a partial proportional odds model will be used as a sensitivity analysis. As an unadjusted sensitivity analysis, the monotonic association between the 0- to 6-hour change in relative renin ratio and 28-day organ failure–free days will also be summarized using the Spearman rank correlation coefficient (ρ) with a 95% CI.

Secondary analyses will include logistic regression models to evaluate the association between renin reduction and 28-day mortality. The interaction between renin reduction and hemodynamic response to AT II will also be explored. The association between early changes in vasopressor requirement (ΔNED) and MAP (ΔMAP) and subsequent survival will first be assessed as continuous variables using a Cox proportional hazards model with a 1-hour landmark. Patients with a decrease in NED at 1 hour (ΔNED<0) will be considered responders according to the clinical vasopressor titration protocol. Among patients with an increase in both NED and MAP (ΔNED>0 and ΔMAP>0), ΔMAP/ΔNED will additionally be explored as a continuous measure of hemodynamic responsiveness and its association with survival. If informative, an appropriate threshold may subsequently be explored to classify patients according to responsiveness, with responder status then evaluated in relation to survival. The same prespecified covariate set will be used in secondary analyses. The association with 28-day mortality will be evaluated using multivariable binary logistic regression. ICU-free days, vasopressor-free days, renal replacement therapy–free days, and invasive mechanical ventilation–free days will be evaluated using proportional odds ordinal logistic regression, with death within 28 days coded as 0 free days. Total NED from 0 to 6 hours will be analyzed using a gamma generalized linear model with a log link. Comparisons of the prognostic performance of renin, lactate, and their product for 28-day mortality will be exploratory and report areas under the receiver operating characteristic curve with 95% CIs.

Sensitivity analyses will be performed to assess the robustness of the results in the presence of missing data. These will include complete case vs available case comparisons and best-case and worst-case scenario analyses to explore the potential range of outcome estimates. This approach will allow us to evaluate the impact of missing data without relying on imputation methods.

Additional exploratory analyses will be conducted to assess the impact of comorbid conditions such as chronic kidney disease or heart failure on the relationship between renin kinetics and treatment response.

For the single primary analysis, a 2-sided α of .05 will be used. To address multiplicity, Holm-adjusted *P* values will be reported within the family of secondary outcome analyses and separately within the family of subgroup interaction tests; both unadjusted and adjusted *P* values will be shown. Other exploratory analyses will be interpreted using effect estimates and 95% CIs without confirmatory claims. Analyses will be conducted using Python (Python Software Foundation).

The amount, pattern, and reasons for missing data will be reported by analysis variable and compared between participants with and without paired renin measurements. The primary model will use participants with observed baseline and 6-hour renin measurements and an observed primary outcome. Participants who die after the 6-hour measurement will remain in the primary analysis and be recorded as having 0 organ failure–free days. Participants who die before the 6-hour measurement cannot have a 0- to 6-hour change in relative renin ratio defined and will be described separately; their observed outcomes will remain in analyses that do not require 0- to 6-hour change in relative renin ratio.

#### Study Dropouts and Missing Data

If the patient or their relatives or legal representatives request a withdrawal from the study or no blood sample for renin concentration is taken before or 6 hours after starting AT II infusion, the patient will be classified as a dropout. The data recorded until consent withdrawal will be stored and used for analysis. We predict a 5% dropout rate for the sample size calculation.

### Data Monitoring

The principal investigator, the coordinating investigator, local investigators, and an international expert are part of the steering committee, by whom the study was designed and all protocol revisions were made.

Access to the documents and data will be granted to the principal investigator, coordinating investigators, auditors, and monitors.

Daily screening for eligible patients will be conducted by local investigators and dedicated intensivists. Data will be collected by the principal investigator, coordinating investigators, and local investigators, and strict adherence to the trial protocol and relevant regulatory requirements will be ensured.

Protocol violations (deviations) will be reported to the principal investigator and discussed with the principal investigator and the coordinating investigators.

### Ethical Considerations

#### Overview

The protocol was approved by the Institutional Medical Ethics Committee of UMC Maribor (UKC-MB-KME-33/24) and the Medical Ethics Committee of the Republic of Slovenia (0120-201/2025-2711-6). The study is registered in a public registry at ClinicalTrials.gov (NCT06539234). It was endorsed by the Slovenian Society of Intensive Medicine.

Any significant protocol revisions will be approved by the Medical Ethics Committee of the Republic of Slovenia, communicated to the sponsor, communicated to *JMIR Research Protocols*, and updated on the ClinicalTrials.gov registry.

The study will be conducted according to the Declaration of Helsinki and Good Clinical Practice guidelines and will comply with the principles of the General Data Protection Regulation national and international regulatory requirements.

#### Informed Consent

All eligible patients or their relatives or legal representatives will receive study information and an invitation to participate. Written informed consent will be obtained after sufficient time for independent decision-making. Participants or representatives may withdraw at any time for any reason without consequences.

Consent will be documented on a signed paper form, with copies retained by the patient and stored securely in a designated folder at each participating center. The form details study basics, voluntary participation, data confidentiality, and withdrawal rights. All documents are ethics committee approved (Multimedia Appendix 1).

#### Data Management

Clinical data will be collected from the time of screening to the discharge of the patient from the hospital. Until discharge from the ICU, data will be recorded daily. Every study participant will be identified by a unique study number, and all collected data will be anonymized: any identifying information, including name, date of birth, and medical record numbers, will be removed.

Additional assurance of the quality of data acquisition will include manual audits of data entry carried out by the study principal investigator for a randomly selected subset of enrolled patients (by generating a random list of 25 numbers between 1 and 121 to select which patients to audit).

#### Confidentiality

Research reports are strictly prohibited from identifying participants by their names. These reports are solely intended for research purposes. Efforts will be made to maintain the confidentiality of participants’ personal medical data.

### Ancillary and Posttrial Care

Patients will be treated according to established guidelines in all other aspects of treatment outside the 6-hour study intervention.

Patients or their legal representatives will be provided, within the informed consent form, with the contact details of a physician who can address any questions that may arise during the course of the study or in the event of a serious adverse reaction to vasopressor therapy. All adverse effects related to the use of vasopressor agents (including AT II) will be managed individually according to the internal protocol of each hospital.

### Dissemination Policy

Findings will be disseminated to health care professionals, participants, and the public via publication in peer-reviewed journals, conference presentations, and social media without any publication restrictions. The final manuscript will be drafted by the primary investigators.

## Results

The research was funded in December 2024 by an institutional research grant from UMC Maribor. It received full ethics approval in July 2025, after which data collection commenced (on August 1, 2025). As of July 2026, a total of 70 patients had been enrolled. Data collection is expected to be completed in 2027, after which the results will be analyzed and published.

## Discussion

### Expected Findings

The TENSINTEST study is designed to determine whether an early decrease in the relative renin ratio within 6 hours of AT II initiation identifies patients with distributive shock who are most likely to benefit from AT II therapy (Textbox 2). We hypothesize that a negative trend in the relative renin ratio will be associated with more 28-day organ failure–free days, more ICU-free days, reduced vasopressor requirements, and lower mortality. We further hypothesize that early changes in renin will predict mortality more accurately than lactate alone and that the combined renin-lactate biomarker will provide superior prognostic performance compared with either biomarker alone. The study aims to identify patients who are likely to benefit from AT II while outlining nonresponders in whom continued AT II treatment is unlikely to improve outcomes, allowing earlier discontinuation of AT II and consideration of alternative therapies.

Textbox 2. **Outcomes.**

**Primary outcome measure**

- Association between 0‐ to 6-h change in relative renin ratio (ΔRRR = RRR_6 h_ − RRR_0 h_) and 28-day organ failure–free days (without vasopressors, mechanical ventilation, and renal replacement therapy [RRT])

**Secondary outcome measures**

- Association between ΔRRR and 28-d intensive care unit–free days
- Association between ΔRRR and vasopressor-free days
- Association between ΔRRR and cumulative dose of vasopressors expressed in norepinephrine-equivalent dose (NED)
- Association between ΔRRR and RRT-free days
- Association between ΔRRR and invasive mechanical ventilation–free days
- Determination of responders and nonresponders based on the calculation of the ratio between change in mean arterial pressure between 0 and 1 h of the protocol and change in total vasopressor NED between 0 and 1 h of the protocol
- Comparison of the prognostic performance of renin and lactate at 0 and 6 h for 28-d mortality
- Determination of whether the product (renin × lactate) at 0 and 6 h predicts mortality
- Determination of whether the change in total NED at baseline and 1 h predicts mortality

A post hoc ATHOS-3 analysis showed lower renin in AT II vs placebo groups [19], mirrored in post–cardiac surgery studies [18] and a prospective feasibility trial comparing AT II to norepinephrine assessing consent, adherence, infusion duration, MAP maintenance, and adverse outcomes [28]. Patients exhibited varied AT II responses, with different phenotypes for hyperresponders, responders, or nonresponders, probably linked to RAAS dysfunction and hemodynamic changes [7,8]. The 6-hour interval is derived from a study by Gleeson et al [21], who determined that renin change rates over 6 hours differed significantly between survivors and nonsurvivors, enabling rapid laboratory assessment. The RAAS underpins circulatory homeostasis; renin levels increase with poor perfusion, increased sympathetic drive, and hypoxia, outperforming lactate in tracking hemodynamic changes [21,22]. As lactate and renin capture distinct pathologies, their product may superiorly classify responders vs nonresponders over either alone.

This is an emerging and clinically relevant area of research, with an increasing number of studies investigating biomarkers that may predict response to AT II therapy. However, our study has a larger planned sample size and is being carried out in more centers than other ongoing studies [29,30]. These other studies randomize patients to AT II use, whereas this study will only evaluate patients to whom AT II is prescribed as part of routine clinical care as a third-line vasopressor, which better matches the current clinical use, and will also evaluate AT II use in all types of vasodilatory shock, not only septic shock. If our hypotheses are confirmed, TENSINTEST could support a biomarker-guided approach to AT II therapy, avoiding unnecessary treatment in nonresponders, optimizing vasopressor use, improving organ recovery, and shortening ICU length of stay.

### Limitations

Due to the relatively small sample size and exclusion of patients with a component of cardiogenic shock, the results of our study might not be useful for a general population of patients with shock who are severely ill. As no similar prospective study has been conducted so far, sample size estimation could not be optimal.

A further limitation is the absence of a control arm, which prevents us from determining whether the prognostic significance of changes in renin is specific to AT II exposure or would also apply to patients treated with other vasopressors.

An additional limitation is that patients are enrolled only after reaching the institutional threshold for third-line vasopressor therapy (norepinephrine ≥0.3 μg per kilogram per minute plus vasopressin of 0.03 IU per minute). Consequently, our findings may not be directly applicable to patients with earlier stages of vasodilatory shock, in whom earlier initiation of AT II is increasingly being investigated. An additional consequence of AT II being used as a third-line vasopressor in patients who are severely ill is that we expect to observe high mortality and a potential beneficial effect of AT II in patients who are less severely ill, and use of AT II at lower cumulative doses of vasopressors might not be observed. Future studies should determine whether the predictive value of early renin kinetics extends to patients receiving AT II at lower vasopressor requirements.

## Supplementary material

10.2196/102437 Multimedia Appendix 1 Details about trial settings.

10.2196/102437 Checklist 1 SPIRIT checklist.

## Abbreviations

APACHE II: Acute Physiology and Chronic Health Evaluation II
AT II: angiotensin II
ATHOS-3: Angiotensin II for the Treatment of High-Output Shock 3
EDTA: ethylenediaminetetraacetic acid
ICU: intensive care unit
MAP: mean arterial pressure
NED: norepinephrine-equivalent dose
RAAS: renin-angiotensin-aldosterone system
SPIRIT: Standard Protocol Items: Recommendations for Interventional Trials
TENSINTEST: Angiotensin II Stress Test
UMC: University Medical Centre
