# Supplementary material for: Renin Kinetics and Hemodynamic Response During Treatment of Vasoplegic Shock With Angiotensin II (TENSINTEST): Protocol for a Multicenter Observational Study
Source: JMIR Res Protoc. 2026 Nov 9;15:e102437. doi: 10.2196/102437 (PMC13747218; doi:10.2196/102437)
Supplement: Supplementary file 2 [file resprot-v15-e102437-s001.docx]

**Supporting information**

**S1. Details about trail setting – ICU**

Trail setting includes two academic hospitals (University Medical Centre Maribor and University Medical Centre Ljubljana) with two general surgical ICUs, one specialized surgical ICU for cardiovascular surgery and one medical ICU. It then includes 2 medical ICUs in community hospitals, 1 surgical ICU in community hospital and one surgical ICU in the oncology institute (see the list below).

- 1. Surgical ICU in the Department of anaesthesiology, intensive therapy and pain management, University Medical Centre Maribor, Maribor, Slovenia
- 2. Medical ICU, Department of Medical Intensive Care Unit, University Medical Centre Maribor, Maribor, Slovenia
- 3. Surgical ICU in Department of intensive care unit, Department of Anaesthetics and Surgical Intensive Care, Ljubljana University Medical Centre, Ljubljana, Slovenia.
- 4. Surgical ICU in Cardiovascular intensive care unit, Department of Anaesthetics and Surgical Intensive Care, Ljubljana University Medical Centre, Ljubljana, Slovenia.
- 5. Medical ICU, Department of Medical Intensive Care Unit, General Hospital in Celje, Slovenia
- 6. Medical ICU, Department of Medical Intensive Care Unit, General Hospital in Novo Mesto, Slovenia
- 7. Surgical ICU, Department of Intensive Care, General Hospital in Slovenj Gradec, Slovenia
- 8. Surgical ICU, in the Department of Anaesthesiology and Intensive Care, Divison of Surgery, Institute of Oncology, Ljubljana, Slovenia

**S2. Informed Consent**

**INFORMED CONSENT TO PARTICIPATE IN A MEDICAL RESEARCH STUDY**

**DATA SECTION**
**Patient Information / Patient label**

**The patient has received a copy:** YES	NO

**Special requirements** (e.g., different language / alternative communication method):

**Responsible physician providing the explanation:** _________________________________

**Study Information**
**Title of the study:** *Angiotensin II Stress Test (TENSINTEST). Renin kinetics during the treatment of vasoplegic shock with angiotensin II in relation to the hemodynamic response to angiotensin II therapy.*

**Organizing institution, unit, address, contact:**
University Medical Centre Maribor, Department of Anesthesiology, Intensive Care and Pain Management, Tel. +386 (0)2 321 1571, and Department of Intensive Internal Medicine, Tel. +386 (0)2 321 247, Ljubljanska 5, 2000 Maribor, Slovenia.

**EXPLANATORY SECTION**

**Title of the study:** *Angiotensin II Stress Test (TENSINTEST). Renin kinetics during the treatment of vasoplegic shock with angiotensin II in relation to the hemodynamic response to angiotensin II therapy*

**Principal Investigator and contact details:** Assoc. Prof. Dr. Andreja Möller Petrun, MD. Department of Anesthesiology, Intensive Care and Pain Management. University Medical Centre Maribor, Ljubljanska 5, 2000 Maribor
Email: andreja.moellerpetrun@ukc-mb.si
Tel.: +386 (0)2 321 1571

**Purpose and course of the study**

Shock is a life-threatening condition that leads to insufficient delivery of oxygen and nutrients to the body’s cells. It can cause multiple organ failure (kidney, intestinal, liver, heart, and lung failure, coagulation disorders with bleeding and clot formation) or even death. Shock is typically treated in the intensive care unit (ICU). One of its main features is low blood pressure, which is managed with specific drugs that constrict blood vessels. These medications are collectively known as vasopressors. The most commonly used vasopressors are norepinephrine and vasopressin, while in recent years angiotensin II has also been introduced.

Each vasopressor acts through a different mechanism, but their overall effect is similar: they constrict blood vessels, redistribute blood flow to vital organs such as the heart and lungs, and raise blood pressure.

Angiotensin II is a naturally occurring vasopressor in the human body and a key component of the renin–angiotensin–aldosterone system, which regulates blood pressure and electrolyte balance. During shock, this system becomes dysregulated. Reduced angiotensin II levels contribute to low blood pressure and impaired organ perfusion, while renin concentrations rise as the body unsuccessfully attempts to produce more angiotensin II.

Synthetic angiotensin II is available as a therapeutic option when norepinephrine and vasopressin alone are insufficient to achieve the target blood pressure. Studies suggest that patients receiving angiotensin II require lower doses of norepinephrine and vasopressin to reach the same target blood pressure. Combining three vasopressors in patients unresponsive to one or two agents therefore allows for lower doses of each individual drug, potentially reducing adverse effects such as immune suppression or cardiac rhythm disturbances with norepinephrine, or intestinal ischemia with high-dose vasopressin.

Angiotensin II itself raises blood pressure and may reduce the risk of kidney injury due to shock. However, like other vasopressors, it can cause excessive vasoconstriction and tissue ischemia in less vital areas such as the fingers, toes, and intestines. Other possible adverse effects include increased heart rate and thrombocytopenia (low platelet count). An increased risk of blood clots has not yet been confirmed or excluded beyond the risk already associated with shock itself. Not all patients respond to angiotensin II with a sufficient increase in blood pressure.

To identify which patients benefit most from angiotensin II, renin measurement has been suggested. Renin is a physiological enzyme responsible for generating angiotensin II when needed. Under normal conditions, angiotensin II suppresses renin release, maintaining balance. This mechanism is disrupted during shock. Elevated renin levels are observed in severely ill shock patients, reflecting the body’s unsuccessful attempt to produce more angiotensin II. High renin concentrations are also an indicator of shock severity. Importantly, many patients with high renin levels respond well to angiotensin II therapy, often showing a subsequent decrease in renin concentration. However, this is not the case for all patients.

We will therefore conduct a prospective study including patients in shock who meet the criteria for angiotensin II administration. The purpose of the study is to determine the relationship between renin concentration before and 6 hours after initiation of angiotensin II therapy and the patient’s clinical outcome. This may help identify which patients benefit most from angiotensin II treatment, maximizing effectiveness while minimizing adverse effects.

The study will be conducted at the Unit for Intensive Care in Surgical Specialties, the Unit for Cardiothoracic Perioperative Intensive Care —both part of the Department of Anesthesiology, Intensive Care and Pain Management— and the Department of Intensive Internal Medicine, all at University Medical Centre Maribor. All patients will receive standard ICU treatment; the only additional procedure will be measurement of renin concentration 6 hours after starting angiotensin II infusion, obtained from a blood sample already drawn for routine clinical analyses.

Participation in the study will not expose patients to any additional risks.

**Duration and location of the study**

The study will be conducted at the Department of Anesthesiology, Intensive Care and Pain Management and at the Department of Intensive Internal Medicine, University Medical Centre Maribor, from approximately August 2024 to August 2026.

**Voluntary participation**

**Your participation in this study is voluntary and may be withdrawn at any time. Withdrawal will not affect the quality of medical care you receive or the attitude of the healthcare team toward you.**

**CONSENT SECTION**

**Personal consent of the patient:**

I, the undersigned ______________________________________________ (surname and first name of the patient in block letters), have received clear and comprehensible information regarding the study: *Angiotensin II Stress Test (TENSINTEST). Renin kinetics during the treatment of vasoplegic shock with angiotensin II in relation to the hemodynamic response to angiotensin II therapy.*

I understand how my safety will be ensured during the study and that I may request additional information at any time and receive it. I have also been informed that I may withdraw my consent at any point without the need to provide justification, and that discontinuation of participation will not in any way affect the quality of medical care I would otherwise receive.

By signing this form, I voluntarily confirm my willingness to participate in the study. I also consent to the use of my demographic and medical data in anonymized form for scientific purposes.

This form is signed in the presence of the investigator.

**Place, date, and time:** _________________________________________________

**Signature of patient:** _________________________________________________

**Name and surname of investigator:** _________________________________________________

**Signature of investigator:** _________________________________________________

**CONSENT BY LEGAL REPRESENTATIVE OR OTHER AUTHORIZED PERSON (if the patient is not capable of making decisions)**

I, the undersigned ____________________________________________________(surname and first name of the patient’s representative in block letters),

_____________________________________________________(relationship to the patient),

on behalf of __________________________________________ _______________(surname and first name of the patient in block letters),

confirm that I have received clear and comprehensible information regarding the study: *Angiotensin II Stress Test (TENSINTEST). Renin kinetics during the treatment of vasoplegic shock with angiotensin II in relation to the hemodynamic response to angiotensin II therapy.*

I understand how patient safety will be ensured during the study and that I may request additional information at any time and receive it. I have also been informed that I may withdraw consent at any point without the need to provide justification, and that withdrawal will not affect the patient’s ongoing medical care.

By signing this form, I voluntarily consent to the patient’s participation in the study and agree that demographic and medical data may be used in anonymized form for scientific purposes. This form is signed in the presence of the investigator.

**Place, date, and time:** _________________________________________________

**Signature of representative:** ____________________________________________

**Name and surname of investigator:** ______________________________________

**Signature of investigator:** ______________________________________________

**SUBSEQUENT PERSONAL CONSENT OF THE PATIENT**

(to be signed once the patient regains decision-making capacity, if inclusion in the study was initially approved by a legal representative or family member)

I, the undersigned __________________________________________ (surname and first name of the patient in block letters), confirm that I have received clear and comprehensible information regarding the study: *Angiotensin II Stress Test (TENSINTEST). Renin kinetics during the treatment of vasoplegic shock with angiotensin II in relation to the hemodynamic response to angiotensin II therapy.*

At the time of my inclusion in the study, I was unable to make decisions regarding participation due to the nature of my illness, and consent was therefore provided on my behalf by my legal representative or relative. I understand how my safety will be ensured during the study and that I may request additional information at any time and receive it. I have also been informed that I may withdraw my consent at any point without the need to provide justification, and that withdrawal will not in any way affect the medical care I may otherwise receive.

By signing this form, I voluntarily confirm my willingness to participate in the study, following the prior consent provided by my legal representative or relative. I also consent to the use of my demographic and medical data in anonymized form for scientific purposes. This form is signed in the presence of the investigator.

**Place, date, and time:** _________________________________________________

**Signature of patient:** _________________________________________________

**Name and surname of investigator:** ______________________________________

**Signature of investigator:** ______________________________________________

**S3. Relative renin ratio**

For the purpose of standardization of renin levels, since different methods are used for obtaining renin plasma concentration, we will report changes in renin level levels as a relative renin ratio (RRR), which is the renin plasma concentration either at baseline or at six hours after treatment with AT II, divided by upper normal concentration of plasma renin (50 mU/L)

$RRR= \frac{renin concentration at specific time (baseline or after six hours of ATII)}{upper normal concentration of renin (50\frac{mU}{L})}$
